# Fraction from Wax Apple [*Syzygium samarangense* (Blume) Merrill and Perry] Fruit Extract Ameliorates Insulin Resistance via Modulating Insulin Signaling and Inflammation Pathway in Tumor Necrosis Factor α-Treated FL83B Mouse Hepatocytes

**DOI:** 10.3390/ijms13078562

**Published:** 2012-07-10

**Authors:** Szu-Chuan Shen, Wen-Chang Chang, Chiao-Li Chang

**Affiliations:** 1 Department of Human Development and Family Studies, National Taiwan Normal University, No. 162, Sec. 1, Heping East Road, Taipei 10610, Taiwan; 2 Graduate Institute of Food Science and Technology, National Taiwan University, P.O. Box 23-14, Taipei 10672, Taiwan; E-Mails: d99641001@ntu.edu.tw (W.-C.C.); carrieli700@hotmail.com (C.-L.C.)

**Keywords:** wax apple, insulin resistance, glucose uptake, western blot, inflammation

## Abstract

Inflammation is associated with the development of insulin resistance in Type 2 diabetes mellitus. In the present study, mouse FL83B cells were treated with tumor necrosis factor-alpha (TNF-α) to induce insulin resistance, and then co-incubated with a fraction from wax apple fruit extract (FWFE). This fraction significantly increased the uptake of the nonradioactive fluorescent indicator 2-[*N*-(7-nitrobenz-2-oxa-1,3-diazol-4-yl) amino]-2-deoxy-d-glucose (2-NBDG) in insulin resistant cells. Western blot analysis revealed that, compared with the TNF-α-treated control group, FWFE increased the expression of the insulin receptor (IR), insulin receptor substrate-1 (IRS-1), protein kinase B (Akt/PKB), phosphatidylinositol-3 kinase (PI3K), and glucose transporter 2 (GLUT-2), and increased IR tyrosyl phosporylation, in insulin resistant FL83B cells. However, FWFE decreased phosphorylation of c-Jun *N*-terminal kinases (JNK), but not the expression of the intercellular signal-regulated kinases (ERK), in the same cells. These results suggest that FWFE might alleviate insulin resistance in TNF-α-treated FL83B cells by activating PI3K-Akt/PKB signaling and inhibiting inflammatory response via suppression of JNK, rather than ERK, activation.

## 1. Introduction

Diabetes mellitus (DM) is one of the major chronic diseases to affect humans. In 2010, there were approximately 220 million DM patients worldwide and this number is expected to increase to 346 million by 2030. More than 95% of all cases of diabetes are Type 2 DM [1]. Diabetes mellitus can cause neuronal and tissue damage, leading to impaired organ function and tissue necrosis. The etiology of Type 2 diabetes mellitus is associated with insulin resistance in peripheral tissues or insufficient secretion of insulin in pancreas β cells [2]. This insulin resistance results from decreased insulin sensitivity in tissues.

The liver is one of the major organs to regulate carbohydrate metabolism. Insulin modulates the release and uptake of glucose in the liver according the requirements of the body [3]. Lower insulin action or insulin resistance has been associated with lower insulin-stimulated activities of enzymes such as glycogen synthase and hexokinase and a reduced ability of insulin to activate a variety of elements of the insulin signaling system, such as tyrosine phosphorylation of the insulin receptor (IR) and insulin receptor substrate 1 (IRS-1), *etc*. [4].

Diabetes mellitus is a chronic disease characterized by inflammation. Recent evidence suggests that inflammation is associated with insulin resistance [5]. Long term exposure to a diet high in sugar or fat leads to an imbalance in oxidative stress; thus stimulating the immune system to secrete cytokines, including TNF-α, IL-6, and IL-10 [6]. It is believed that these inflammatory factors can reduce insulin signaling by phosphorylating IRS-1 on several serine residues that might lower the ability of IRS-1 to transduce the insulin signaling system [4].

Wax apple [*Syzygium samarangense* (Blume) Merrill and Perry] belongs to the Myrtaceae family and is among the fruits of economic importance in Asia and Taiwan. Its leaves, roots, bark, and fruit all have potential medical applications. Some Myrtaceae plants have reportedly been used as medicinal herbs for the treatment of bronchitis, asthma, diabetic mellitus, and inflammation syndromes [7]. They exert potent free radical scavenging, antioxidation, antimutation, and anticancer activities [8]. Our previous study identified that extracts from guava (*Psidium guajava* L.) leaves displayed antihyperglycemic ability in Type 2 diabetic rats and recognized quercetin as the major active component in the extracts [9,10]. The leaves of wax apple also contain abundant phytochemicals, including ellagitannins, flavanones, flavonol glycosides, proanthocyanidins, anthocyanidins, triterpenoids, chalcones, and volatile terpenoids [11–16]. Wax apple fruit has reportedly demonstrated antihyperglycemic activity in allxan-induced (Type 1 DM) diabetic mice [15]. However, no study on the association between wax apple and insulin resistance (Type 2 DM) has been reported. Moreover, the mechanism of wax apple regarding attenuating insulin resistance is far from clear.

The present study aimed to investigate the effect of a fraction from wax apple fruit extract (FWFE) on alleviating insulin resistance in TNF-α treated insulin resistant FL83B mouse hepatocytes. The uptake of a glucose analog in FL83B cells was evaluated. The expressions of insulin signal-associated proteins, glucose transporter 2 (GLUT-2), and inflammation-associated proteins in FL83B cells were analyzed using Western blotting. These results could enhance the understanding of anti-hyperglycemic mechanisms and the application of wax apple in Type 2 DM.

## 2. Results and Discussion

### 2.1. The Characterization of FWFE

**Figure 1:**
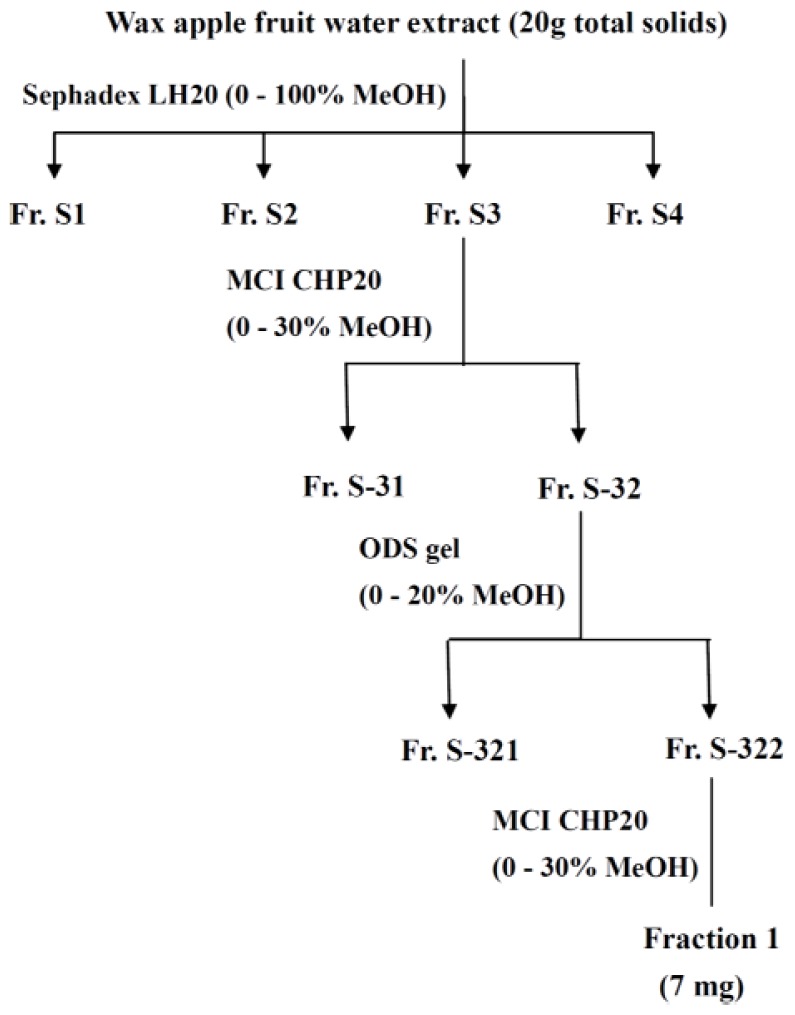
The flow chart for fractionation of wax apple fruit water extract.

The flow chart for fractionation of wax apple fruit water extract is shown in Figure 1. Wax apple fruit water extract freeze-died powder was redissolved and separated by column chromatography to obtain fractions with different polarities. Polar components, such as phenolic acids and glycosides of many flavonoids are usually extracted with water, alcohol, or a mixture of the two. In comparison with ethanol, methanol has higher polarity and extraction efficiency [17]. Polar components were considered to be more functional. We used a series of columns, including sephadex LH 20, MCI CHP20, and ODS gel with MeOH-H_2_O solution to separate wax apple fruit extract into fractions based on polarity. The components with weak polarity were ignored. Therefore, FWFE might be characterized as a high polarity fraction.

Wax apple has been reported to contain abundant phytochemicals [11–16]. The recovery of FWFE in wax apple fruit water extract after filtration, concentration, and freeze-drying was 0.035%. The freeze-dried FWFE was analyzed to contain a high level of phenolic compounds (401.1 ± 6.4 mg gallic acid equivalents/g dried weight) and flavonoids (80.9 ± 3.5 mg quercetin equivalents/g dried weight).

### 2.2. Effects of FWFE on Glucose Uptake in Insulin Resistant FL83B Mouse Hepatocytes

Tumor necrosis factor-α may be associated with an insulin-resistant state in animals. The administration of exogenous TNF-α into an *in vitro* cell model and into an *in vivo* animal model may induce insulin resistance. TNF-α reportedly interferes with insulin signal transduction and subsequently influences with carbohydrate metabolism in cells and tissues [18,19]. The possible mechanisms for TNF-α to impair insulin signal transduction involve the down-regulation of insulin receptor (IR) and insulin receptor substrate-1 (IRS-1) expressions, the inhibition of tyrosyl phosphorylation of IR and IRS-1, the increase in serine/threonine phosphorylation of IRS-1, the decrease in the activities of insulin receptor kinase and protein tyrosine phosphatases (PTPs), and the inhibition of insulin-stimulated glucose transporter [19,20].

**Figure 2:**
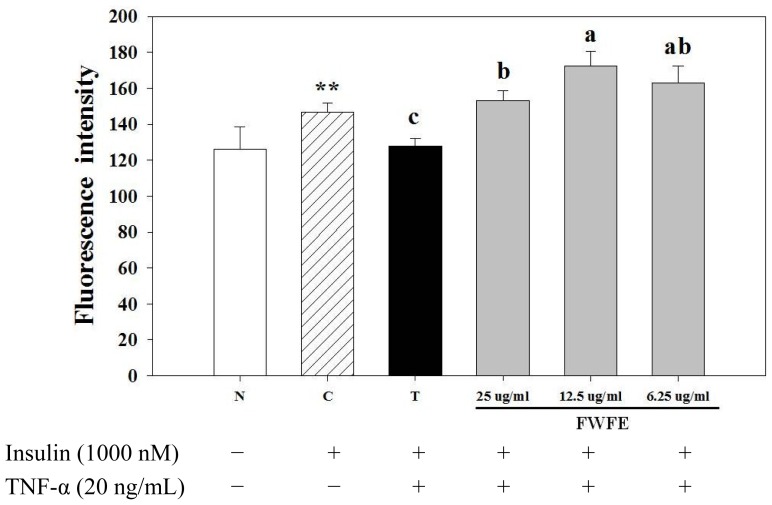
Effects of FWFE on glucose uptake in TNF-α-treated FL83B mouse hepatocytes. ** significantly different (*p* < 0.01) from N (normal) group. Letters a,b indicate significant differences at 5% level. N: normal group, cells incubated with F-12K medium. C: control group, cells incubated with F-12K medium containing 1000 nM insulin. T: treated group, TNF-α-induced insulin resistant cells.

As shown in Figure 2, the addition of TNF-α decreased the uptake of glucose in normal FL83B hepatocytes. However, FWFE increased glucose uptake in FL83B hepatocytes as compared with the TNF-α-treated control group (*p* < 0.01). Resurreccion-Magno *et al*. [15] previously reported the hypoglycemic bioactivity of wax apple fruit in alloxan-induced (Type 1 DM) diabetic mice and suggested that chalcones, an intermediate product of isoflavone biosynthesis and polyphenol derivatives in plants, and their derivates, are the major anti-diabetic components. However, no literature regarding the effect and mechanism of wax apple with insulin resistance (Type 2 DM) has been reported. In addition, our present study found that FWFE contained high amounts of phenolics and flavonoids. The anti-oxidative effect of phenolics in diabetic animals has been reported [21]. Flavonoids are known to be involved in the healing process of free radical-mediated diseases, including diabetes [22]. Thus, the improvement effect of FWFE on glucose uptake in insulin resistant FL83B mouse hepatocytes might be partially the result of the anti-oxidative contribution of these phytochemicals.

### 2.3. Insulin Receptor Expression and IR Tyrosyl Phosphorylation

In the normal situation, insulin binds to the IR in cell membranes to activate IR tyrosine kinase and triggers a series of insulin signal transduction responses in cells. The IR is a glycoprotein tetramer located in the cell membrane and is constructed from 2 α subunits and 2 β subunits. The combination of insulin and the IR α subunits leads to alterations in the structure morphology. The aggregation of 2 α subunits results in the auto-phosphorylation of the 2 β subunits [23]. The phosphorylated IR promotes tyrosyl phosphorylation of IRS-1,2,3 to activate downstream molecules and subsequently modulate blood glucose levels [24,25].

**Figure 3:**
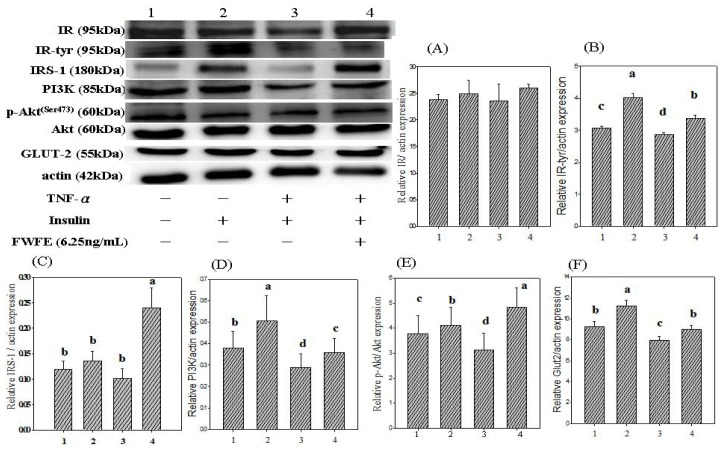
Effect of FWFE on TNF-α-induced inhibition of insulin signals in FL83B cells. FL83B cells were incubated in serum-free F12K medium, with or without added TNF-α (20 ng/mL), incubated at 37 °C for 5 h, transferred to another serum-free F12K medium with or without insulin (1000 nM), FWFE (6.25 μg/mL), and then incubated for an additional 30 min. The basal level of glucose uptake was evaluated by incubating cells with the serum-free F12K medium. The relative expressions of IR, IR-tyr, IRS-1, PI3K (p85), p-Akt/Akt, and GLUT-2 in each treatment group were calculated using actin as the standard. Letters a~d indicate significant differences at 5% level.

In the present study, the addition of 1000 nM insulin (positive control group) to normal FL83B cells increased IR expression by 4.6% and IR tyrosyl phosphorylation by 31.1% (Figure 3A,B). Previously, Shahid and Hussain [26] reported that 100 nM insulin markedly increased IR expression and IR tyrosyl phosphorylation in normal FL83B hepatocytes. Other cytokines, such as TNF-α and IL-1, also reportedly inhibited IR expression and IR tyrosyl phosphorylation in 3T3-L1 adipocytes and HepG2 carcinomar cells [27]. In the present study, FWFE increased IR expression by 10.1%, and IR tyrosyl phosphorylation by 18.1%, in FL83B hepatocytes as compared with the TNF-α-treated control group (*p* < 0.05) (Figure 3A,B). The results suggested that FWFE increases IR expression and IR tyrosyl phosphorylation, and consequently activates the downstream signaling molecules in insulin resistant FL83B cells.

### 2.4. Insulin Receptor Substrate Expression

The insulin receptor substrates are cellular signaling carrier proteins involved in the modulation of intracellular bioinformation and recognize messages from insulin receptors. Insulin receptor substrate-1 is the major insulin-like growth factor-I receptor (IGF-IR). The expression of IRS-1 increases with the development of DM complications, such as liver cancer and pancreatic cancer [28]. Insulin stimulates phosphorylation of IRS-1 at multiple signaling molecules, including P85, Grb2, Nck/Crk, Syp/Fyn, and SHP2 [29]. The serine/threonine phosphorylation of IRS-1 subsequently suppresses tyrosine phosphorylation and blocks insulin signaling [30].

Figure 3C shows the effects of FWFE on IRS expression in TNF-α-treated FL83B hepatocytes. The addition of insulin increased IRS-1 expression by 14.8% in insulin resistant FL83B cells. Benito reported the conferring of insulin resistance in IRS-1 gene knockout adipocytes and the liver cells [31]. A previous study reported that TNF-α promoted IRS-1 serine phosphorylation and led to decreased downstream PI3K expression, proposed as the principal cause of insulin resistance [30]. Hotamisligil *et al*. [32] also reported that TNF-α decreased the expression of IR and downstream IRS-1 in myeloid 32D cells. In the present study, TNF-α decreased IRS-1 serine phosphorylation by 26.0% in FL83B hepatocytes as compared with the positive control group (insulin-treated group). However, FWFE increased IRS-1 expression by 238.1% in TNF-α-treated FL83B hepatocytes, indicating the restoration of intercellular insulin signaling.

### 2.5. Phosphatidylinositol-3 Kinase Expression

The stimulation of insulin promotes IR tyrosyl phosphorylation, catalyzes the insulin receptor family, and triggers the activation of 2 major cell growth and nutrient metabolism pathways: the Phophatidylinositol-3 kinase (PI3K) and Mitogen-activated protein kinase (MAPK) pathways [3]. PI3K plays an important role in the insulin transduction pathway. The activation of PI3K is required for the translocation of glucose transporters [33]. A previous study identified suppressed activities of PI3K and its downstream regulating proteins in primary muscle fibroblast cells from diabetic patients [34]. The suppression of PI3K activity might reduce insulin sensitivity and the efficiency of translocation of the glucose transporters, leading to the inhibition of glycogen synthesis-related gene modulation [35,36].

As shown in Figure 3D, insulin increased PI3K expression by 33.3% in normal FL83B cells. In contrast, the expression of PI3K was 56.5% lower in TNF-α-treated hepatocytes than in the insulin-treated group (*p* < 0.05), indicating the induction of insulin resistance in FL83B cells. Treatment with FWFE increased PI3K expression in the insulin resistant FL83B mouse hepatocytes by 21% compared with the TNF-α-treated control group (*p* < 0.05) (Figure 3D) suggesting that FWFE might increase the expression of downstream molecules by increasing the phosphorylation of insulin receptors in insulin resistant FL83B mouse hepatocytes.

### 2.6. Expression of Akt/Protein Kinase B

The enzyme Akt, also known as protein kinase B (PKB), plays a key role in tissue growth. It modulates the translocation of glucose transporters and synthesis of glycogen by activating GSK-3α and GSK-β in cells. Inhibitors of PI3K, such as LY294002 and wortmannin, can suppress the expression of upstream proteins to arrest Akt phosphorylation [27]. Insulin reportedly increases Akt phosphorylation in HepG2 human carcinoma cells [37].

In the present study, the addition of insulin induced a 5.0% increment in Akt serine^473^ phosphorylation in normal FL83B cells (Figure 3E). In contrast, TNF-α-treated FL83B cells displayed a 23.3% decrease of Akt serine^473^ phosphorylation (Figure 3E). More recently, Guitton *et al*. [38] reported that co-incubation with TNF-α markedly suppressed phosphorylated Akt protein expression in primary human blood vessel esoderma cells. In the present study, Akt phosphorylation was 54.7% higher in the FWFE-treated group than in the TNF-α-treated FL83B cells (Figure 3E), suggesting that FWFE might restore PI3K expression and activate the phosphorylation of downstream Akt/PKB in TNF-α-induced insulin resistant FL83B mouse hepatocytes.

### 2.7. Glucose Transporter 2 Expression

At least 12 glucose transporters (GLUTs) that mediate glucose uptake in various cells have been reported [39]. GLUT2 is a glucose-sensitive transporting protein and exists in the liver, pancreas β cells, intestinal mucosal cells, and tubular epithelial cells [40,41]. In the present study, the treatment of TNF-α markedly decreased expression of GLUT2 by 21.5% in FL83B mouse hypatocytes (Figure 3F). GLUT2 expression increased by 13.2% after the treatment of FWFE in the TNF-α-induced insulin resistant FL83B cells (Figure 3F).

Above results suggest that FWFE can indirectly increase GLUT2 levels in the cell membrane and enhance glucose uptake in TNF-α-treated FL83B mouse hepatocytes.

### 2.8. Expression of c-Jun N-terminal Kinases

The c-Jun *N*-terminal kinases (JNK) are proteins involved in the MAPK signal transduction pathway. Both TNF-α or IL-1β can trigger activation of JNK and dephosphorylation of IRS-serine^307^, interfere with adjacent phosphorylation binding sites, inhibit normal phosphorylation of tyrosine, and cause insulin resistance. Animals with JNK1 gene knockout displayed reduced adipose tissues, increased insulin sensitivity, and increased insulin signaling activity in peripheral tissues [42]. Free fatty acids and TNF-α are activators of JNK, and can, thus, promote JNK phosphorylation [43]. The activation of JNK promotes IRS-1 serine/threonine phosphorylation in the PI3K/Akt pathway to down-regulate insulin signal transduction.

**Figure 4:**
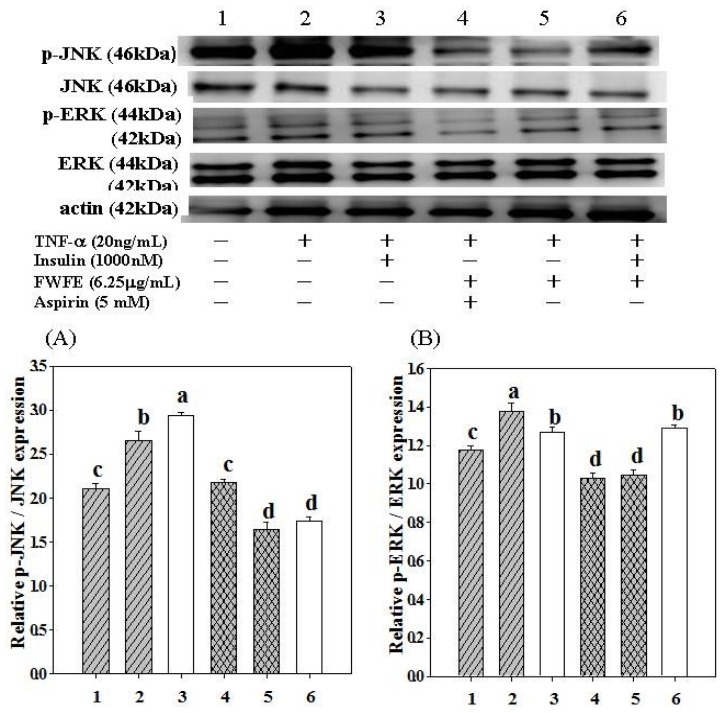
Effect of FWFE on phosphorylated JNK and ERK expression in TNF-α-induced insulin resistant FL83B cells. FL83B cells were incubated in serum-free F12K medium, with or without added TNF-α (20 ng/mL), incubated at 37 °C for 5 h, transferred to another serum-free F12K medium with or without insulin (1000 nM), FWFE (6.25 μg/mL) Aspirin (5 mM), and then incubated for an additional 30 min. The relative expressions of JNK and ERK in each treatment group were calculated using actin as the standard. Letters a~d indicate significant differences at the 5% level.

In the present study, the treatment of TNF-α increased JNK phosphorylation by 26.5% in FL83B mouse hypatocytes (Figure 4A). The addition of insulin plus TNF-α increased JNK phosphorylation by 39.7% in FL83B cells (Figure 4A). The JNK signal transduction pathway, which can also be stimulated by insulin, is highly associated with the syndrome of inflammation [43]. Thus, addition of insulin can increase phosphorylation of JNK. Aspirin is known as an anti-inflammatory drug and was used as a positive control in the present study. It has recently been observed that Aspirin inhibited sepsis-induced JNK activity accompanied by a reduction in IRS-1 serine phosphorylation at Ser^307^ [44]. The addition of Aspirin plus FWFE decreased JNK phosphorylation by 17.7% in TNF-α treated FL83B cells (Figure 4A). The addition of FWFE alone, however, decreased phosphorylation of JNK by 38.2% (Figure 4A). The addition of insulin plus FWFE decreased JNK phosphorylation by 34.5% in TNF-α-treated FL83B cells (Figure 4A). These results from the present study suggest that FWFE effectively inhibits intercellular inflammatory signaling cascades by suppressing JNK phosphorylation and, therefore, might restore insulin signaling in TNF-α treated FL83B cells.

### 2.9. Expression of Extracellular Signal-Regulated Kinases

The ERKs are downstream proteins of several growth factors (such as EGF, NGF, and PDGF) and responsible for regulating cell proliferation, differentiation, and survival. High sugar levels and cytokines (such as IL-6, AngII, TNF-α, IL-6, and IFN-γ) are precipitating factors of DM [45]. Both sugar and cytokines activate membrane ERK and decrease the binding activity of IRS to IR, thus, suppresses phosphorylation of PI3K and interferes with transduction of IR, IRS, and PI3K in insulin signaling.

In the present study, the treatment of TNF-α increased ERK phosphorylation by 17.1% in FL83B mouse hepatocytes (Figure 4B). The addition of insulin and FWFE decreased ERK phosphorylation by 7.9% and 6.6%, respectively, in TNF-α treated FL83B mouse hepatocytes. The addition of Aspirin and Aspirin plus FWFE decreased ERK phosphorylation by 25.3% and 24.2% respectively, with no statistically significant difference in TNF-α treated mouse FL83B cell, suggesting that the ERK signaling cascade is not the major pathway for FWFE to restore intercellular insulin signaling.

Our present study showed that FWFE markedly attenuates insulin resistance by restoring IRS-1 phosphorylation and up-regulation of PI3K-Akt/PKB insulin signaling via the inhibition of JNK (rather than ERK) phosphorylation in TNF-α-treated FL83B mouse hepatocytes.

## 3. Experimental Section

### 3.1. Chemicals and Reagents

Bovine serum albumin (BSA), D-(+)-glucose, insulin, 4-(2-hydroxyethyl)-1-piperazineethanesulfonic acid (HEPES), recombinant mouse TNF-α, 3-(4,5-dimethylthiazol-2-yl)-2,5-diphenyl-tetrazolium bromide (MTT reagent), and F12 Ham Kaighn’s modification (F12K) medium were purchased from Sigma-Aldrich Co. (St. Louis, MO, USA). Fetal bovine serum (FBS) was obtained from Gemini Bio-Products (Woodland, CA, USA). The fluorescent dye 2-(*N*-(7-nitrobenz-2-oxa-1,3-diazol-4-yl)amino)-2-deoxyglucose (2-NBDG) was purchased from Invitrogen (Eugene, OR, USA). All of the chemicals used in this study were of analytical grade.

### 3.2. Wax Apple Fruits

Wax apples [*Syzygium samarangense* (Blume) Merrill and Perry cv. Pink cultivars] were collected during the third week of fruit blooming in Shuang-Hsi Township, Taipei County during 2010, July.

### 3.3. Preparation of Extract

The wax apples were washed, drained, weighed, sliced, and freeze-dried. Each 1 g aliquot of the dried material was extracted with 6 mL distilled water (1:6, w/v) at 4 °C for 72 h and filtered through cheese cloth. The filtrate was filtered twice through Whatman No. 1 filter paper and then centrifuged at 4700 g for 20 min. The supernatant was vacuum-concentrated using a rotary evaporator at a temperature below 40 °C. The concentrate was freeze-dried into wax apple fruit water extract powder and stored at −80 °C.

### 3.4. Fractionation of the Extract

The methods were based on those proposed by Chang and Shen [46] with minor modifications (Figure 1). Briefly, an aliquot of 20 g reconstituted wax apple fruit extract was run through a Sephadex LH-20 (St. Louis, MO, USA) column with 0% to 100% MeOH (500 mL) as the eluent. The fractionated eluates in the collector from single experiments were then pooled into S1, S2, S3, and S4 fractions according to the order of elution and the thin layer chromatography (TLC) profile. A silica gel precoated plate (Kieselgel 60 F254, 0.20 mm, Merck, Darmstadt, Germany) with a mobile phase of benzene:ethylformiat:formic acid = 1:5:2 was used for TLC analysis. The TLC plate was atmosphere-dried then sprayed with 10% sulfuric acid containing 5% FeCl_3_ to elucidate the distribution of compounds in the column eluate. Fraction S3 was run through the MCI-gel CHP 20P (2 cm × 30 cm) (Mitsubishi Chemical Industries, Tokyo, Japan) using gradient elution with MeOH-H_2_O (0:100, 10:90, 20:80, and 30:70; 300 mL in each stage) to obtain fractions S-31 and S-32. Fraction S-32 was run through the Sephadex LH-20 column (2 cm × 30 cm) using gradient elution with H_2_O-MeOH to obtain fractions S-321 and S-322. Fraction S-322 was further chromatographed over an MCI-gel CHP 20P column using gradient elution with H_2_O-MeOH to obtain fraction A. Every 3 mL of fraction A was collected. Adjacent fractions were pooled based on the TLC profile and then freeze-dried as a powder (Fraction 1, 7 mg). This fraction from wax apple fruit extract (FWFE) was used as the specimen in further experiments.

### 3.5. Determination of Phenolics and Flavonoids Content

Total phenolics content was determined by the Folin-Ciocalteau method [47], and expressed as gallic acid equivalents. Total flavonoids content was measured using the method of Zhishen *et al*. [48], and expressed as quercetin equivalents.

### 3.6. Cell Culture

The experiments were performed on mouse liver FL83B cells; a hepatocyte cell line deriving from a fetal mouse (15 days to 17 days). The cells were incubated in F12K containing 10% FBS and 1% penicillin and streptomycin (Invitrogen Corporation, Camarillo, CA, USA) in 10-cm Petri dishes at 37 °C and 5% CO_2_. Experiments were performed on cells that were 80% to 90% confluent.

### 3.7. Induction of Insulin Resistance Using TNF-α

The methods were adapted from Chang and Shen [46] with minor modifications. Briefly, the FL83B cells were seeded in 10-cm dishes and then incubated at 37 °C for 48 h to achieve 80% confluence. Serum-free F12K medium containing 20 ng/mL recombinant mouse TNF-α was then added before incubating for 5 h to induce insulin resistance.

### 3.8. Cell Preparation

The FL83B cells were incubated in F12K medium without (basal) or with TNF-α (20 ng/mL) for 5 h to induce insulin resistance. The cells were then transferred to another F12K medium containing 5 mM glucose, without (basal) or with 5.0 μg/mL insulin and 6.25 ng/mL FWFE, and incubated for 3 h at 37 °C. Assay of glucose uptake was then performed.

### 3.9. Uptake of Fluorescent 2-NBDG in Mouse FL83B Hepatocytes

The glucose uptake assay was performed using the methods reported by Chang and Shen [43] with minor modifications. Briefly, the FL83B cells were detached from the dish using trypsin treatment and then suspended in 1200 μL Kerbs-Ringer biocarbonate buffer containing 1 μM insulin. Each 172 μL aliquot of the cell suspension was transferred to an eppendorf tube and co-incubated with 20 μL 1 μM insulin and 8 μL 2-NBDG (the final concentration of 2-NBDG is 200 μM) in a 37 °C water bath for 1 h in the dark. The reaction was stopped on ice. The cell suspension was then centrifuged at 3000 g (4 °C) for 5 min to discard the supernatant. The pellet was washed with phosphate buffered saline (PBS) and centrifuged 3 times before being suspended in 1 mL PBS. The fluorescent intensity of the cell suspension was evaluated using flow cytometry (FACScan, Becton Dickinson, Franklin Lakes, NJ, USA) at an excitation wavelength of 488 nm and an emission wavelength of 542 nm. The intensity of fluorescence reflected the uptake of 2-NBDG in the cells.

### 3.10. Protein Extraction from Cells

After pre-incubation in serum-free F12K medium with or without TNF-α at 37 °C for 5 h, FL83B cells were transferred to another serum-free F12K medium with/without insulin or FWFE for 3 h. The medium was removed. The cells were washed twice with ice cold PBS and then lysed in ice cold lysis buffer containing 20 mM Tris-HCl (pH 7.4), 1% Triton X-100, 0.1% SDS, 2 mM EDTA, 10 mM NaF, 1 mM PMSF, 500 μM sodium orthovanadate, and 10 μg/mL antipain. Cell lysates were sonicated four times every 5 s with ice cooling, and then centrifuged (13,000 g, 20 min) to recover the supernatant. The supernatant was removed as the cell extract and stored at −80 °C for further use.

### 3.11. Determination of Protein Concentration

The protein concentration in the cell extract was determined using Bio-Rad protein assay.

### 3.12. Western Blot Analysis

Aliquots of the supernatant, each containing 50 μg protein, were used to evaluate the expression of IR, tyrosyl-phosphorylated insulin receptor (IR-tyr), IRS-1, intercellular ERK, phosphorylated ERK (p-ERK), JNK, and phosphorylated JNK (p-JNK). Aliquots of the supernatant containing 30 μg protein were used to evaluate expression of PI3-kinase, Akt/PKB, phosphorylated protein kinase B (p-Akt/p-PKB), and GLUT-2. The samples were subjected to 10% dodecyl sulfate polyacrylamide gel electrophoresis (SDS-PAGE). The protein spots were electrotransferred to a polyvinylidene difluoride membrane. The membrane was incubated with block buffer (PBS containing 0.05% Tween-20 and 5% w/v nonfat dry milk) for 1 h, washed with PBS containing 0.05% Tween-20 (PBST) 3 times, and then probed with 1:1000 diluted solutions of anti-IR-tyr, anti-PI3K, anti-Akt/PKB, anti-GLUT-2, anti-ERK, and anti-p-ERK antibodies (Cell Signaling Technology, Beverly, MA, USA), 1:1000 diluted solution of anti-IRS-1 antibody (Gene Tex, Irvine, CA, USA), 1:2000 diluted solutions of anti-IR and anti-p-Akt/p-PKB antibodies (Abcam, Cambridge, UK), 1:1000 diluted solutions of anti-JNK and anti-p-JNK antibodies (Epitomics, Burlingame, CA, USA) overnight at 4 °C. The intensity of the blots probed with 1:2000 diluted solution of mouse monoclonal antibody to bind actin (BD Biosciences, Franklin Lakes, NJ, USA) was used as the control to ensure that a constant amount of protein was loaded into each lane of the gel. The membrane was washed three times for 5 min each time in PBST, shaken in a solution of HRP-linked anti-mouse IgG or anti-rabbit IgG secondary antibody, washed a further three times for 5 min each time in PBST, and then exposed to the enhanced chemiluminesence (ECL) reagent (Millipore) according to the manufacturer’s instructions. Autoradiography was performed on Fuji medical X-ray film (Fuji, Tokyo, Japan).

### 3.13. Statistical Analysis

The data were analyzed using one-way ANOVA and Duncan’s new multiple range test. A *p* value < 0.05 was considered significant.

## 4. Conclusions

**Figure 5:**
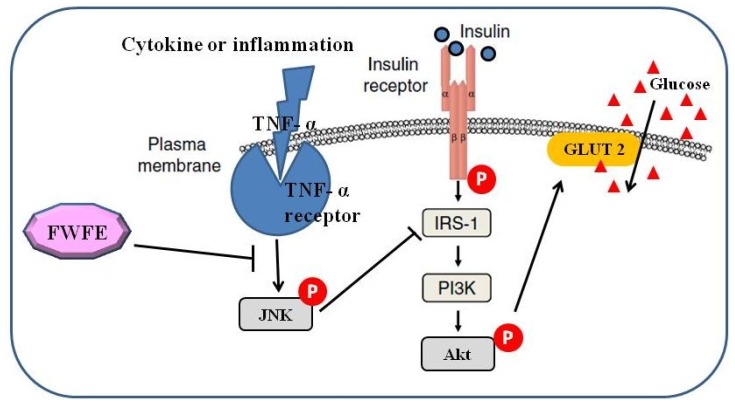
Postulated mechanism of FWFE on modulating insulin signaling and inflammation pathway in TNF-α-induced insulin resistant FL83B cells.

This present study investigated the effects of FWFE on glucose uptake, and the expression of insulin and inflammatory signal transduction-related proteins in TNF-α-induced insulin resistant FL83B mouse hepatocytes. We postulate that FWFE can alleviate insulin resistance via inhibiting the intracellular JNK inflammatory signaling cascades, restoring the PI3K-Akt/PKB insulin signaling pathway, and then enhancing glucose uptake in TNF-α-treated FL83B mouse hepatocytes (Figure 5). Therefore, FWFE might have potential use in the development of anti-diabetic drugs, health foods, and dietary supplements. Further investigation on the purification and identification of active compounds in FWFE is currently underway in our laboratory.

## Abbreviations

Akt/PKB: protein kinase B
BSA: bovine serum albumin
DM: diabetes mellitus
ERK: extracellular signal-regulated kinases
FBS: fetal bovine serum
F12K: F12 Ham Kaighn’s modification
FWFE: fraction from wax apple fruit extract
GLUT2: glucose transporter 2
HEPES: 4-(2-hydroxyethyl)-1-piperazineethanesulfonic acid
IGF-IR: insulin-like growth factor-I receptor
IR: insulin receptor
IRS-1: insulin receptor substrate-1
IR-tyr: tyrosyl-phosphorylated insulin receptor
JNK: c-Jun *N*-terminal kinases
MAPK: Mitogen-activated protein kinase
2-NBDG: 2-[*N*-(7-nitrobenz-2-oxa-1,3-diazol-4-yl) amino]-2-deoxy-D-glucose
p-Akt/p-PKB: phosphorylated protein kinase B
PBS: phosphate buffered saline
p-ERK: phosphorylated ERK
PI3K: phosphatidylinositol-3 kinase
p-JNK: phosphorylated JNK
SDS-PAGE: dodecyl sulfate polyacrylamide gel electrophoresis
TLC: thin layer chromatography
TNF-α: tumor necrosis factor-alpha
